# Medicine, Pathology and Therapeutics

**Published:** 1898-06

**Authors:** Albert T. Lytle

**Affiliations:** Buffalo, N. Y.


### Progress in Medical Science.

### MEDICINE, PATHOLOGY AND THERAPEUTICS.

Conducted by ALBERT T. LYTLE, M. D , Buffalo, N. Y.

### PNEUMONIA.

RUSSELL, in a valuable article upon pneumonia (*Medical News*), gives deductions from 134 cases in his private practice. His death-rate was 16.4 per cent. as against 18.1 per cent. from hospital statistics, demonstrating the large fatality of this acute disease. He had seventy-three cases in children under ten years with twelve deaths (16.4 per cent.) His experience would negative the contagiousness of pneumonia by any of the ordinary means; the duration was very irregular, being one to fifteen days. He believes that dulness on percussion and weakened breath-sounds over the affected area are far more constant and safe signs of beginning consolidation than the crepitant râle which is so often absent. In children he finds a fear of falling at the very beginning of the disease, which to him has become almost pathognomonic, the child will be still with high fever and if raised will clutch the nurse and express great fear of falling; they after complain of pain in the stomach in place of in the side. He is guarded in prognosis, emphasising that the course of the disease and recovery therefrom depends upon two important characteristics, the local lesion and the systematic intoxication and that these do not necessarily show either direct or indirect relations; consequently judgment cannot be accurately formed. He says of treatment, "In a disease in which, in fully one-half of all cases, treatment is apparently ineffectual and in which recovery is frequent even when treatment is not attempted, the cases not being seen by a physician, one must speak with great diffidence, especially as regards drugs. I believe that much good may be accomplished by proper treatment, and that it is criminal to permit a patient to be wholly without treatment. In my opinion, treatment consists in rest and care of the heart. Rest is certainly the most essential point in treatment." To obtain rest, beside putting the patient in bed, he allays restlessness by the judicious use of morphine in hypodermic doses of grains 1-8, and makes rest as absolute as possible. He carefully watches the heart and uses strychnin, digitalis and whisky when it flags. He deprecates the routine use of jackets and poultices, but he has had immediate and striking relief from pain and consequent embarrassment of breathing by the application of a poultice, either over the affected area or over the whole chest. He uses cold bathing to control temperature and believes the local application of ice to be theoretically correct, but he has not been encouraged to use it by his experience except in cases of hyperpyrexia.

---

### DELAYED RESOLUTION IN PNEUMONIA.

ALFRED STENGEL concludes a paper in the *Therapeutic Gazette* upon the treatment of delayed resolution in pneumonia as follows: First, that in cases of only slight tendency to delay, manifested by moderate dulness and persistent broncho-vesicular breathing, systematic breathing exercises are of the greatest importance. Secondly, that when considerable dulness persists, active counter-irritation should be practised and tonics and stimulants administered. Thirdly, that the production of aseptic abscesses (by the hypodermic injection of turpentine, one to two cubic centermeters) may be useful; the cases in which this has been tried are too few to warrant absolute conclusions and the treatment is too painful for general application.

---

### OBSTINATE DROPSIES.

TYSON, in the treatment of obstinate dropsies, whether due to heart, kidney or liver, advises practically the same general treatment consisting of rest in bed; restriction of the diet to about one-half to one quart of milk in twenty-four hours in divided doses, and until patient complains of hunger, when the food is gradually increased to meet the demands; free purgation, using large doses of alkaline salts in concentrated solution, preferably in the morning, or compound jalap powder, or calomel and soda in large doses. After free catharsis, he begins the use of tincture digitalis in doses of 10 minims, four times a day, which he administers for 48 hours, when if no effect is produced he increases the dose until one drachm is given in 24 hours, then if no effect is obtained he changes the drug; he watches the pulse carefully during the digitalis administration and if it reaches 60 he discontinues it. He uses digitalis intermittently to avoid toxicity and to get effects. As the action of nitroglycerine is to dilate arterioles, he often uses it with digitalis, but he prefers theobromin which occasionally is superior to digitalis and which he gives to the amount of 45 grains in 24 hours, in divided doses, at three-hour intervals and for a period of six days if diuresis is effected ; diuretin, a compound containing theobromin, does not seem to have been satisfactory. After theobromin he uses spartem, then strophanthus and then calomel, squill and digitalis. He occasionally uses elaterium, but does not like its uncertain action. He calls upon the skin by means of baths, Turkish, Russian, hot wet packs and hot bottle packs; for drugs he uses pilocarpin in hypodermic injection not exceeding one quarter of a grain, combating dangerous edemia by use of atropin, 1-100 to 1-50 grains, hypodermatically. As a last resort he performs paracentesis, puncture or incision for the purpose of drainage. He relates cases of dropsy from heart, from kidney and from liver to illustrate his methods as above.—*Ther. Gaz.*

---

### ATYPICAL RHEUMATISM.

SINGER (*Med. News*) in discussing a typical rheumatic arthritis before the medical club of Vienna says, "Typical rheumatism is characterised by swelling of the joints and disorders of the skin, heart and serous membranes, which are favorably influenced by salicylates. In 1885, Immerman called attention to certain cases of neuralgia of the trigeminus, beginning with fever and accompanied with swelling of the joints and heart symptoms, and which were favorably influenced by salicylates. Edlefsen called this an embryonic form of polyarthritis, and today we include in it certain cases of sciatica. That chorea is to be classed among the atypical forms of rheumatism is shown by the symptoms in joints and endocardum, as well as by certain bacteriological discoveries. Moreover, polyneuritis has much in common with polyarthritis which it often follows. The post-rheumatic muscular atrophies, which owe their existence to polyneuritis, must be included in this classification. Herpes labialis and herpes zoster may also be considered as atypical manifestions of rheumatism. Therefore the joint symptoms should be regarded as the most striking rather than the most universal manifestation of rheumatism. The most reliable symptom is fever, which is common to all forms and often extends beyond the period in which there are symptoms in the joints." The etiology, he claims, lies in an infection of the blood.

